# Insights into cyanobacterial alkane biosynthesis

**DOI:** 10.1093/jimb/kuab075

**Published:** 2021-10-29

**Authors:** Humaira Parveen, Syed Shams Yazdani

**Affiliations:** Microbial Engineering Group, International Centre for Genetic Engineering and Biotechnology, New Delhi 110067 India; Microbial Engineering Group, International Centre for Genetic Engineering and Biotechnology, New Delhi 110067 India; DBT-ICGEB Centre for Advanced Bioenergy Research, International Centre for Genetic Engineering and Biotechnology, New Delhi 110067, India

**Keywords:** Cyanobacteria, Alkane, Biofuels, Microbial Engineering, CRISPR

## Abstract

Alkanes are high-energy molecules that are compatible with enduring liquid fuel infrastructures, which make them highly suitable for being next-generation biofuels. Though biological production of alkanes has been reported in various microorganisms, the reports citing photosynthetic cyanobacteria as natural producers have been the most consistent for the long-chain alkanes and alkenes (C15–C19). However, the production of alkane in cyanobacteria is low, leading to its extraction being uneconomical for commercial purposes. In order to make alkane production economically feasible from cyanobacteria, the titre and yield need to be increased by several orders of magnitude. In the recent past, efforts have been made to enhance alkane production, although with a little gain in yield, leaving space for much improvement. Genetic manipulation in cyanobacteria is considered challenging, but recent advancements in genetic engineering tools may assist in manipulating the genome in order to enhance alkane production. Further, advancement in a basic understanding of metabolic pathways and gene functioning will guide future research for harvesting the potential of these tiny photosynthetically efficient factories. In this review, our focus would be to highlight the current knowledge available on cyanobacterial alkane production, and the potential aspects of developing cyanobacterium as an economical source of biofuel. Further insights into different metabolic pathways and hosts explored so far, and possible challenges in scaling up the production of alkanes will also be discussed.

## Introduction

The rising demand for energy due to an exponential increase in human population has led to the over-exploitation of non-renewable energy resources, resulting in the shrinking of fossil fuel reserves at a fast pace. The injudicious use of fossil fuels has resulted in increased emission of CO_2_ in the atmosphere, culminating in global warming (Naduthodi et al., 2018).

In recent years, there has been an increasing trend of producing biofuel from various organic sources using different methods (Santos-Merino et al., 2019). However, a corresponding scale up to match the ever-increasing demand for fuel has not been achieved. Thus, an economical and sustainable source of biofuel production is invaluable. Another aspect of biofuel production is the environmental impact of the proposed source and methodology to be used, which should be taken into consideration for greater goals of green and clean energy (Jaroensuk et al., 2019).

Alkanes (C15-C17) are considered as the most suitable candidate for biofuel because of their high cetane number (Wang et al., 2013), a measure for combustion speed and ignitibility (Jiménez-Díaz et al., 2017). It also has gained attention due to its compatibility with the existing liquid fuel infrastructures (Ling et al., 2013). Further properties like high-energy density and hydrophobicity, which are prerequisites of jet fuels, diesel, and gasoline, make it an ideal candidate for drop-in biofuel (Shakeel et al., 2018).

Many microorganisms have been reported to produce alkanes, however, cyanobacteria are found to be the most promising candidate for generating biofuels such as alkanes (Wang et al., 2013). Cyanobacteria have broad geographical distribution and are diverse in terms of genetic composition, morphology, and growth habits. Certain cyanobacterial strains show a remarkable ability to fix atmospheric nitrogen using specialised cells called heterocyst (Lau et al., 2015). The most valuable feature of these tiny green organisms that attracted the researchers is the production of diverse secondary metabolites (Xue & He, 2015) that are under exploration for various purposes, including the discovery of novel pharmacological inhibitors (Singh et al., 2011). They can be found in diverse ecological habitats, varying from terrestrial, marine to freshwater. Apart from generating oxygen, cyanobacteria also contribute significantly to the internal hydrocarbon cycle of the oceans by producing 2–540 pg alkanes per mL per day, therefore, they might play a significant role in the biogeochemical cycle (Lea-Smith et al., 2015).

Biofuel production by the recycling of released carbon dioxide by cyanobacteria is a novel approach that allows liberty from fossil fuel dependency (Arias et al., 2020). In some of the cyanobacteria, alkane biosynthesis is essential for normal physiological growth (Lea-Smith et al., 2016), however, precise mechanism is not well understood (Coates et al., 2014). Further, cyanobacteria also show the ability to tolerate long-chain alkanes (Xie et al., 2017). Apart from bacteria and cyanobacteria, alkane production has been reported in other micro-organisms like algae, yeasts, and fungi (Wang et al., 2013), which might help to protect them against environmental hazards and support optimal growth (Valentine & Reddy, 2015).

Certain characteristics of cyanobacteria make them promising host for generating biofuels such as alkanes (Wang et al., 2013). They have a shorter life cycle and fast growth rate when compared to other photosynthetic organisms such as plants and algae. They are genetically tractable and have more fuel-efficient oxygenic photosynthesis than microalgae and higher plants (Luan et al., 2019). Cyanobacteria can grow photo-autotrophically, and unlike heterotrophic microbes such as bacteria and yeast, they can fix atmospheric carbon and some can fix atmospheric nitrogen too. Further, cyanobacteria can make use of various organic carbon sources for growth (Vijay et al., 2019) and show unique ability of producing hydrocarbon derived from fatty acid (Coates et al., 2014). Their sturdy physiology, excellent photosynthetic capacity, and ability to grow in varying temperatures, coupled with their eminent genetic tractability, and low nutrient requirements, fascinated researchers to study and engineer them for the production of biofuels (Gibbons et al., 2018; Vijay et al., 2019). In terms of biomass production, cyanobacteria give a higher yield than terrestrial biofuel crops (Huang et al., 2010). Despite many advantages that cyanobacteria have in terms of a preferred microbial system for biofuel production, it has some limitations as follows:

1. The metabolic and gene regulatory networks in cyanobacteria are not well understood and well characterised.
2. When compared to yeast or *Escherichia coli*, in which synthetic biology experiment can be done in days, cyanobacteria require significant time period ranging from weeks to months, due to a relatively slower growth rate (Yu et al., 2014).

Cyanobacteria are having a peculiar thylakoid membrane that harbours a complete set of respiratory enzymes as well as photosynthetic apparatus (Cooley & Vermaas, 2001). Alkane synthesis occurs primarily in the thylakoid membrane (Wang et al., 2020). In freshwater cyanobacteria, predominantly detected hydrocarbons are heptadecane, while marine cyanobacteria produce pentadecane (Shakeel et al., 2015). Cyanobacteria produce a higher amount of even chain fatty acid as compared to odd chain fatty acid, the latter is produced in negligible amounts (Park et al., 2020). While the majority of the cyanobacteria produce odd chain alkane, the production of even chain alkanes has also been reported in some cyanobacterial strains (Gelpi et al., 1970) (Table 1). It is possible that these may be a product of an unknown pathway or have been misidentified (Coates et al., 2014).

**Table 1.** Comparative Distribution of Alkanes in Cyanobacterial Species

Alkanes	Cyanobacteria	% of total hydrocarbon	Reference
Tetradecane	*Spirulina platensis*	34.61	Ozdemir et al. (2004)
Pentadecane	*Anacystis sp.*	16, 21	Han et al. (1968), and Winters et al. (1969)
	*Microcoleus lyngbyaceus*	2	Winters et al. (1969)
	*Microcystis aeruginosa*	18	Dembitsky and Srebnik (2002)
	*Oscillatoria sp.*	9	Winters et al., (1969)
	*Plectonema terebrans*	6	Winters et al., (1969)
	*Osillatoria woronichinii*	93	Dembitsky and Srebnik (2002)
	*Spirulina platensis*	3.20	Ozdemir et al. (2004)
Hexadecane	*Anacystis sp.*	2.6, 5	Han et al. (1968) and Winters et al. (1969)
	*Lyngbya sp.*	4	Winters et al. (1969)
	*Microcoleus sp.*	4	Winters et al. (1969)
	*Microcystis aeruginosa*	5	Dembitsky and Srebnik (2002)
	*Plectonema terebrans*	3	Winters et al. (1969)
	*Spirulina platensis*	2.18	Ozdemir et al. (2004)
	*Nostoc commune*	1.5	Dembitsky and Srebnik (2002)
	*Osillatoria woronichinii*	2	Dembitsky and Srebnik (2002)
	*Prochloron sp*.	1.2	Perry et al. (1978)
	*Synechococcus bacillaris*	2	Blumer et al. (1971)
	*Trichodesmium erythraeum*	2	Han et al. (1968)
Heptadecane	*Most cyanobacteria*	Most prominent	Dembitsky and Srebnik (2002) and Winters et al. (1969)
Octadecane	*Anacystis cyanea*	13	Gelpi et al. (1970)
	*Calothrix*	30.9	Paoletti et al. (1976)
	*Microcystis aeruginosa*	6	Walsh et al. (1998)
	*Synechocyctis* UTEX 2470	19	Sakata et al. (1997)
	*Lyngbya sp*.	1	Gelpi et al. (1970)
	*Anacystis nidulans*	2.5	Dembitsky and Srebnik (2002)
Nonadecane	*Calothrix sp.*	10.4	Paoletti et al. (1976)
	*Microcystis aeruginosa*	2	Walsh et al. (1998)
	*Spirulina platensis*	1.9	Dembitsky and Srebnik (2002)

## Alkane Production by Cyanobacteria and Its Physiological Effects

A majority of cyanobacterial strains has been found to be capable of synthesising alkanes (Klähn et al., 2014) (Table 1). In *Nostoc punctiforme*, during the exponential growth phase, alkanes were found to be stored in lipid droplets (LDs), with heptadecane (C17) found to be produced maximum (0.77% ± 0.06% of DCW) (Peramuna et al., 2015; Peramuna & Summers, 2014). Further, alkanes are primarily localised in the thylakoid membrane and cell membrane, probably due to their hydrophobic and non-polar nature (Lea-Smith et al., 2016). There are limited studies available that can explain the precise physiological role played by alkanes in cyanobacteria and the advantages they may provide to the host. Nonetheless, few recent studies have shed light on the possible role alkanes could play. Lea-Smith et al. reported that the deletion of the acyl ACP reductase (AAR) gene involved in alkane biosynthesis in *Synechocystis* sp. PCC6803 and olefin synthase gene from *Synechococcus elongatus* PCC7002 led to altered cell morphology and meaningful phenotypic differences in comparison to wild type (Lea-Smith et al., 2016). The differences reported were increase in cell size, diminished growth, and cell division defects. This suggests alkanes might play a role in determining cell morphology and helps normal cell division. However, photosynthetic rates were comparable between both the mutant strain and wild type. Still, the difference was observed in the energy transfer between soluble light harvesting phycobilisome complex and membrane-bound photosystems. Therefore, alkane might control the redox balance and the reductant partitioning in photosynthesis as reported by the Berla group (Berla et al., 2015). In another study involving the generation of knock out of aldehyde-deformylating oxygenase (ADO)-type pathway in model cyanobacterial species, *Synechocystis* sp. PCC6803, the mutant deficient in alkane synthesis was shown to exhibit altered phenotype and increased cyclic electron flow (CEF) at low temperature (∼20ºC) for maintaining redox balance of electron transport chain (ETC). Genome-scale modelling data suggested that the higher CEF pressurises the cells to use less energy-efficient pathway, which in turn leads to the lower quantum efficiency of photosynthesis and result in slower growth (Berla et al., 2015). It was also suggested that alkanes are essential for maintaining proper membrane fluidity and flexibility (Arias et al., 2020; Lea-Smith et al., 2016). Further, data suggest that alkanes are essential for cyanobacteria to tolerate abiotic stresses including cold temperature (20°C) (Berla et al., 2015) and salt (Yamamori et al., 2018). However, while a number of impacts caused by hydrocarbons on cells are known, the precise mechanism underlying through which they translate these effects *in vivo* remains enigmatic (Knoot & Pakrasi, 2019).

A comparative transcriptomic analysis was performed recently for the cyanobacterium (*N. punctiforme*) harbouring alkane-overproducing plasmid and control plasmid (Arias et al., 2020). The analysis suggested that 177 genes were upregulated and 121 genes were downregulated. The majority of genes identified were known to be involved in regulating cellular stress. Based on their roles, 16 upregulated genes were chosen for overexpression in alkane-overproducing strain. Intriguingly, it was found that alkane production was reduced significantly. This suggests that the genes involved in cellular stress might alarm the cells towards the overproduced alkane and act as negative feedback regulators of alkane production (Arias et al., 2020).

Alkane overproduction does not lead to cell damage, however, its precursor aldehyde, when accumulates, is cytotoxic and causes cell bleaching. Recently, the alkane degradation pathway was discovered in which it was proposed that cyanobacterial ADO and aldehyde dehydrogenase (ALDH) was involved in alkane biodegradation to maintain lipid and redox homeostasis. When ROS (reactive oxygen species) generation was induced by high flux light source, ADO, and ALDH together converted the alkane to fatty acid, which can be used later to regenerate acyl chains of damaged lipids and maintain redox homeostasis via scavenger ROS (Qiao et al., 2020).

Until now, the highest reported production of alkanes in the native host is 0.12% of dry biomass (Coates et al., 2014). Genetic engineering provides a meaningful approach for increasing alkane production in cyanobacteria, enabling the use of alkanes as a drop-in fuel (Savakis & Hellingwerf, 2015) (Table 2).

**Table 2.** Engineering Strategies for Alkane Production in Cyanobacteria

Alkane	Host strain	Genetic engineering	Specific yield	Reference
Heptadecane	*Synechocystis* sp. PCC6803	Overexpression of two copies of native AAR and ADO in both slr0168 and slr1556 loci	11 mg/g DCW	Wang et al. (2013)
Heptadecane	*N. punctiforme* PCC73102	Co-overexpression of extra copies of AAR and ADO encoded by Npun_F1710/Npun_F1711 and lipase encoded by Npun_F5141	130 mg/g DCW	Peramuna et al. (2015)
Heptadecane	*Anabaena* sp. PCC7120	Overexpression of AAR and ADO from *Aphanothece* *Halophytica*	1.3 mg/g DCW	Kageyama et al. (2015)
Pentadecane Heptadecane	*Synechococcus* sp. PCC7002	Overexpression of AAR and ADO from *S. elongatus* PCC7942	7.5 mg/g DCW	Knoot and Pakrasi (2019)
Heptadecane	*Synechococcus* sp. NKBG15041c	Overexpression of AAR and ADO from *S. elongatus* PCC7942	4.2 μg/g of DCW	Yoshino et al. (2015)

DCW: dry cell weight.

### Pathway of Alkane Production

**Fig. 1.**
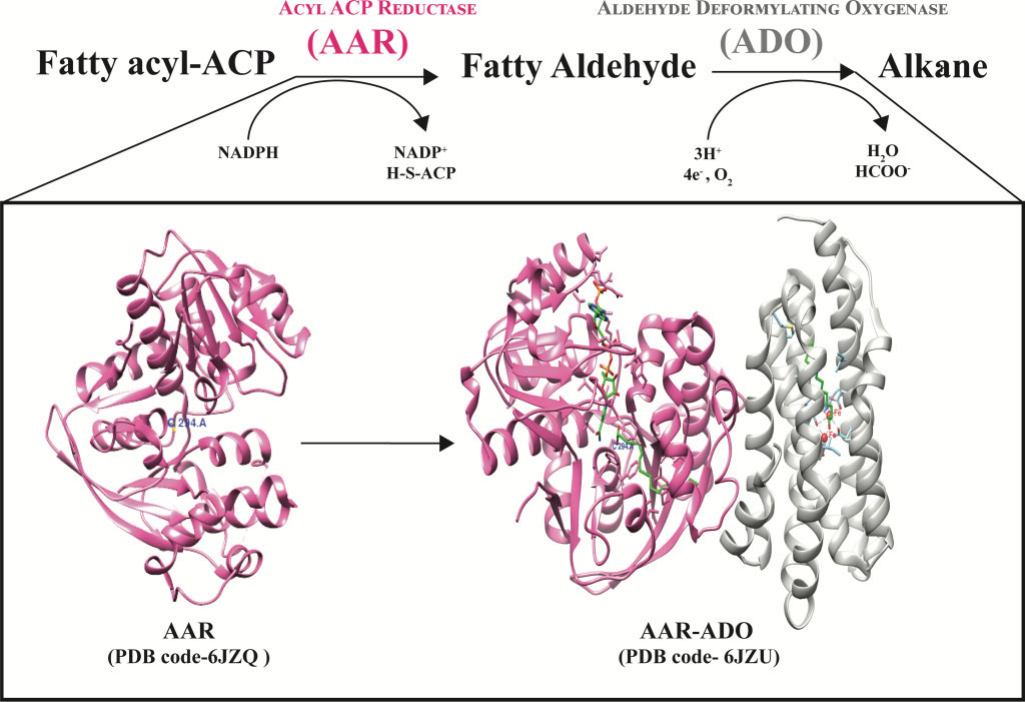
Schematic representation of the alkane biosynthesis pathway in cyanobacteria. Two enzymes were required from fatty acyl ACP to alkane—acyl ACP reductase (AAR) and aldehyde-deformylating oxygenase (ADO). AAR converts fatty acyl ACP to fatty aldehyde using NADPH, and subsequent oxidation of fatty aldehyde to alkane is catalysed by ADO using reduced ferredoxin as reductant.

In the mid-20th century, it was found that cyanobacteria can naturally produce alkane (Knoot & Pakrasi, 2019), but the underlying mechanism or pathway involved was not identified until recently. The pioneering work by the Schirmer group, in which they had performed subtractive genome analysis between alkane-producing and non-producing cyanobacteria, and identified 17 genes that were specifically present in alkane-producing cyanobacteria and absent in non-alkane-producing cyanobacteria (Schirmer et al., 2010). Among the identified genes, the majority were hypothetical in nature, besides two genes that were found to be coding for two crucial enzymes that led to the discovery of the alkane biosynthesis pathway. The two enzymes were AAR and ADO that synthesise alkane using fatty acyl ACP as a substrate (Schirmer et al., 2010) (Fig. 1). Other than cyanobacteria, AAR enzyme homologues are also present in humans, plants, algae, etc.; however, in contrast, ADO is exclusively present only in cyanobacteria (Liu et al., 2013).

Alkane biosynthesis in cyanobacteria is a two-step conversion process which follows the n-1 rule (Waugh & Marsh, 2014). The first step involves the AAR enzyme which catalyses the reduction of fatty acyl ACP into fatty aldehyde using NADPH and Mg^2+^ as a cofactor (Liu et al., 2013) and activated by K^+^ (Bergman & Siewers, 2016; Warui et al., 2015). As fatty aldehydes are toxic to the cells (Gibbons et al., 2018), it further leads to decarboxylation or deformylation into alkane and formate with one carbon less using ADO enzyme (Kaiser et al., 2013). ADO belongs to a family of ferritin-like non-heme di-iron oxygenase which requires oxygen and four electrons for its reducing activity (Arias et al., 2020). One oxygen atom and aldehyde hydrogen are retained by the co-product formate (Jia et al., 2015). *In vitro* studies reported that during the reduction process, ferredoxin (Fd) and ferredoxin-NADP^+^ reductase (FNR) act as an ETC and electrons are transferred from NADPH to FNR followed by Fd and then to ADO that ultimately uses the electrons to carry out the conversion of fatty aldehyde to alkane (Zhang et al., 2013). But under *in vivo* condition, reduced ferredoxin is easily made available in the photosynthetic cell directly by PSII, which can act as an electron donor for ADO activity. However, some reports suggest that ADO is a very slow enzyme with the highest turnover reaches to ∼1 min^–1^ (Andre et al., 2013). Also, AAR protein is prone to aggregation when overexpressed in *E. coli*, leading to poor *in vivo* and *in vitro* catalytic activities (Kudo et al., 2019; Sharma et al., 2021). ADO from *N. punctiforme* and AAR from *S. elongatus* PCC7942 were found to be most efficient amongst various homologues tested (Kudo et al., 2016; Zhu et al., 2018). Electrostatic interaction plays an important role in the interaction between AAR and ADO which helps AAR to efficiently deliver substrate to ADO (Arias et al., 2020; Chang et al., 2020). The team also discovered a site E201 which is important for ADO and AAR interaction (Chang et al., 2020). Further molecular understating of this mechanism has been provided by structural studies. The recently published crystal structure of AAR_apo_ (PDB code 6JZQ) from *S. elongatus* PCC7942 and AAR-ADO complex with ligand (PDB codes 6JZU, 6JZY, and 6JZZ), provides some crucial insight into the elusive biochemistry of the process and helps in the identification of key residue for complex formation (Gao et al., 2020).

ADO and AAR orthologues are found within the genome of all the sequenced cyanobacterial strains that are alkane producers (Kudo et al., 2016; Patrikainen et al., 2017). However, the alkane biosynthesis gene arrangements are different in different cyanobacterial strains. In *Synechocystis* sp PCC6803, both ADO and AAR genes have an independent promoter for their expression, resulting in monocistronic transcript expression (Klähn et al., 2014). While AAR has a single promoter and is located downstream of ADO, the ADO has one proximal and one distal promoter (Klähn et al., 2014). In normal condition, a distal promoter is dominant, while some conditions may exist in which the proximal promoter is dominant (Klähn et al., 2014). In the *S. elongatus* PCC7942, expression of alkane biosynthesis operon is essential as it also includes gene-encoding acetyl CoA carboxylase as part of the polycistronic transcript, which is essential for survival (Klähn et al., 2014; Schirmer et al., 2010).

The AAR amino acid sequence was found to be varying among different cyanobacterial strains with the similarity of 67% (Kudo et al., 2016). When different cyanobacterial AAR and ADO were compared, *N. punctiforme* 73102 ADO (Schirmer et al., 2010) and *S. elongatus* PCC7942 AAR were found to have the highest solubility and activity (Kudo et al., 2016). Further, marine cyanobacterial AAR shows a higher affinity towards C16 fatty acid, while AAR of freshwater cyanobacteria has higher specificity towards C18 fatty acid. The mechanism of difference in affinity for fatty acid carbon chain length is still unknown (Kudo et al., 2019; Shakeel et al., 2015).

### Engineering of Cyanobacteria to Increase Alkane Production

**Fig. 2.**
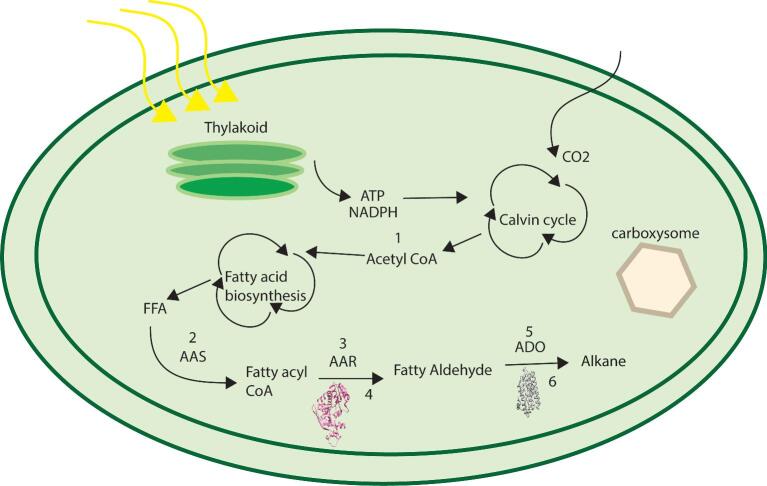
Possible engineering strategies to increase alkane production in cyanobacteria. These are the steps that can be modulated to enhance alkane production: (1) Enhanced production of acetyl CoA, (2) Overexpression of acyl ACP synthetase (AAS) to enhanced acyl ACP Pool, (3) Overexpression of acyl ACP reductase (AAR), (4) Modification of AAR enzyme to improve its activity, (5) Overexpression of aldehyde-deformylating oxygenase (ADO), (6) Modification of ADO enzyme to improve its activity.

Both process and genetic engineering strategies were adopted to enhance the titre of alkane in cyanobacteria. While some researchers have cultured the cyanobacteria under abiotic stress to increase alkane production, others have taken the alternative path of simply overexpressing the genes responsible for alkane production in cyanobacteria and other microorganisms. Moreover, there are several reports available where complex metabolic engineering strategies have been adopted on model cyanobacterial species for increasing the alkane production (Fig. 2, Table 2).

The correlation between abiotic stress and enhanced alkane accumulation in cyanobacteria was first discovered in nitrogen-fixing cyanobacterium *Anabaena sp.* PCC7120, as they doubled their intracellular alkane accumulation upon growth under nitrogen deficiency (Kageyama et al., 2015). Also, alkane production was found to be increased in halotolerant *Anabaena* sp. PCC7120 and *A. halophytica* upon salt stress (Kageyama et al., 2015). However, a negative correlation was found for alkane accumulation in freshwater cyanobacterium *S. elongatus* PCC7942 under similar stress conditions (Yamamori et al., 2018). Therefore, there exists a lack of consensus for using stress to increase alkane production. In *Anabaena cylindrica*, salt stress leads to heterogeneity of alkane carbon chain length (Bhadauriya et al., 2008), but for other cyanobacterial species, it is unknown (Yamamori et al., 2018).

Some cyanobacterial species such as *N. punctiforme*, which normally have LDs, contain fewer alkanes. During the exponential phase, the overexpression of AAR/ADO, lipolytic enzymes and the high flux of light lead to enhanced alkane production, that is, approximately 16-fold higher (∼130 mg/g DCW) as compared to wild-type strain (Peramuna et al., 2015).

Cyanobacterial acyl ACP synthetase (AAS), which helps in recycling released fatty acids, plays an essential role in alkane production (Gao et al., 2012; Wang et al., 2012). Along with AAS, overexpression of AAR and ADO gene simultaneously enhanced acyl ACP pool and increased alkane production to 130% compared to wild type (Wang et al., 2013). Overexpression of the first step of fatty acid biosynthesis catalyses by multi-subunit acetyl CoA carboxylase was also an efficient approach to increase alkane production (Tan et al., 2011).

Alkane production in cyanobacteria can be efficiently enhanced by overexpression of alkane biosynthetic genes in different genomic loci (Wang et al., 2013; Xie et al., 2017). In *Synechocystis*, overexpression of the alkane biosynthetic gene and blocking the competing pathway yield ∼3.6 times compared to wild-type strain, and integration in multiple loci result in ∼26 mg/l of alkane (Wang et al., 2012; Wang et al., 2013). Because of its slow activity, ADO is considered as the rate-limiting enzyme and has become the target for most of the studies for increasing alkane production (Bao et al., 2016).

Apart from the engineering of the cyanobacterial native alkane biosynthesis pathway (Knoot & Pakrasi, 2019), many synthetic pathways were heterologously assembled in cyanobacteria using other organism genes such as fatty acid decarboxylase from *Pseudomonas fluorescens* Pf-5 (UndA, UndB) (Rui et al., 2015; Rui et al., 2014) and fatty acid photodecarboxylase (FAP) from *Chlorella variabilis* for converting the free fatty acid precursor to alkane as an end product yielding around ∼110 mg/l (Sorigué et al., 2017; Yunus et al., 2018).

### Engineering of Heterologous Host for Alkane Production

The alkane biosynthetic pathway can be integrated into heterologous host also for higher alkane production in the non-native hosts such as *E. coli* and yeast (Table 3). The first report on the heterologous expression of the cyanobacterial alkane biosynthetic genes was made by the Schirmer group in *E. coli* host, in which they reported ∼300 mg/l of alkane (Schirmer et al., 2010). Another group employed cyanobacterial alkane biosynthetic genes and used an intracellular pool of free fatty acid directly as a substrate that resulted in the production of ∼5.8 mg/l of a mixture of alkanes/alkenes in *E. coli* (Akhtar et al., 2013; Howard et al., 2013). For the production of chain length-specific alkanes, a variety of enzymes from different sources, such as ADO from *N. punctiforme*, chain-specific thioesterase from *Umbellularia californica*, and fatty acid reductase (FAR) from *Acinetobacter*, were engineered into *E. coli*, yielding medium-chain alkane including 2.21 ± 0.18 mg/g DCW undecane, 1.83 ± 0.12 mg/g DCW tridecane, and 4.01 ± 0.43 mg/g DCW of pentadecane (Yan et al., 2016). Another strategy to increase alkane production is by blocking the competing pathway. Enhancement in alkane production level to ∼250 mg/l was detected when the competing alcohol production pathway was inhibited by deleting the *yqhD* gene which encodes aldehyde reductase, along with overexpression of a gene for transcription factor FadR involved in fatty acid synthesis (Song et al., 2016). Using constraint-based metabolic modelling approach, various potential targets were identified to enhance alkane production and engineered in *E. coli* to yield ∼2.54 g/l (Fatma et al., 2018).

**Table 3.** Engineering strategies for alkane production in heterologous host

Alkane	Host strain	Key enzymes	Resource	Production level	Reference
Pentadecane Heptadecane	*E. coli*	Overexpression of AAR and ADO	*S. elongatus* PCC7942 *N. punctiforme* PCC73102	∼300 mg/l	Schirmer et al. (2010)
Mixture of alkanes	*E. coli*	Overexpression of FAR, ADO	*Photorhabdus luminiscens* *S. elongatus* PCC7942	5.8 mg/l	Howard et al. (2013)
Mixture of alkanes	*E. coli*	Overexpression of FAR, ADO, TE, Sfp	*Mycobacterium marinum* *Prochlorococcus marinus* *E. coli* *Bacillus subtilis*	2 mg/l	Akhtar et al. (2013)
Pentadecane Heptadecane	*E. coli*	Overexpression of AAR, ADO, FadR and the deletion of yqhD	*S. elongatus* PCC7942 *E. coli*	∼250 mg/l	Song et al. (2016)
Mainly pentadecane	*E. coli*	Overexpression of FAR, ADO, FadD, TE	*Acinetobacter sp.* *N. punctiform* PCC73102 *E. coli* *Umbellularia californica*	∼4 mg/g DCW	Yan et al. (2016)
Pentadecane Heptadecane	*E. coli*	Overexpression of AAR, ADO, fd/FNR	*S. elongatus* PCC7942	∼1.3 g/l	Cao et al. (2016)
Pentadecane Heptadecane	*E. coli*	Overexpression of AAR, ADO, G6PDH and deletion of Edd, Pps, LdhA, AceA, PoxB, PlsX	*S. elongatus* PCC7942 *E. coli*	∼2.54 g/l	Fatma et al. (2018)
Mixture of alkanes	*S. cerevisiae*	Overexpression of AAR and ADO	*S. elongatus* PCC7942	∼0.11 mg/l	Kang et al. (2017)
Pentadecane Heptadecane	*S. cerevisiae*	Overexpression of AAR, ADO, fd/FNR and deletion of HFD1	*S. elongatus* PCC7942 *E. coli*	∼20 mg/g DCW	Buijs et al. (2015)
Tridecane Pentadecane Heptadecane	*S. cerevisiae*	Overexpression of CAR, ADO, FFA synthase, *ACC, Adh*5p and deletion of FAA1, FAA4, POX1, HFD1	*M. marinum* *N. punctiforme* *S. cerevisiae*	∼0.8 mg/l	Zhou et al. (2016)

DCW: dry cell weight.

Other strategy that can be employed to increase alkane production is to enhance the catalytic activity of responsible enzymes using the protein engineering approach (Arai et al., 2018). AAR-ADO fusion construct when expressed in *E. coli* leads to ∼5-fold increment in alkane (Rahman et al., 2014). One of the crucial enzymes of the alkane biosynthesis pathway, ADO, was engineered to make it thermostable using a consensus-guided approach for alkane production at higher temperature (Shakeel et al., 2018).

Further studies were done on *Saccharomyces cerevisiae* to produce alkanes in which different aldehyde decarbonylases were compared and found that cyanobacterial ADO is the most suitable enzyme for alkane production in yeast resulting in ∼0.11 mg/l of total alkane (Kang et al., 2017). Alkane production was found to increase around ∼20 μg/g of DCW when the cyanobacterial alkane biosynthesis gene was expressed along with the deletion of hexadecanal dehydrogenase (HFD1) (Buijs et al., 2015). The yeast strain *S. cerevisiae* was engineered to enhance the free fatty acid accumulation along with the expression of alkane biosynthesis gene CAR (*Mycobacterium marinum*) and ADO (*N. punctiforme*) resulted in 0.8 mg/l alkane (Zhou et al., 2016).

### Tools Available for Engineering Cyanobacteria

Cyanobacterial strains usually have natural competency, that is, using homologous recombination they are able to integrate foreign DNA into their genome (Vavitsas et al., 2019). They have neutral sites in their genome, which can be used for the integration of heterologous DNA or gene of interest to multiple loci. This strategy also helps to prevent polar effects on downstream genes, thus maintaining the cell physiology and metabolism (Pinto et al., 2015). Most of the cyanobacterial strains also have endogenous plasmids, that can be used for the expression of the heterologous gene (Hitchcock et al., 2020). Metabolic engineering strategy in cyanobacteria has primarily focussed on increasing production via gene knocked-out, gene knocked-in, overexpression, or differential expression of the gene(s). This can be achieved by using various promoters, ribosome-binding sites (RBS) of varying strength, and other gene regulatory tools such as small RNAs, riboswitches (Oliver et al., 2016; Santos-Merino et al., 2019) or by using high-throughput engineering tool CRISPR/Cas for markerless gene editing (Behler et al., 2018).

In the past few years, remarkable advancement has been made in conventional methodology as well as in the development of the new and efficient genome engineering tools such as different promoters, riboswitches, sRNA, CRISPR/Cas, in addition to using genome modelling for genetic manipulations in cyanobacteria (Xia et al., 2019). By using an appropriate promoter, the gene expression of the target gene can be altered (Till et al., 2020). Constitutive promoters lead to continuous expression of genes while inducible promoters can fine-tune the expression of the specific gene. Some of the known endogenous constitutive promoters which have been widely used are associated with the genes *psbA2, prnpB, pcpcB*, etc. (Zhu et al., 2015). Inducible promoters take advantage when expressing a pathway or a gene that might obtrude a metabolic burden or result in a product that is toxic to the cells (Sun et al., 2018). Some studies have been done to optimise the existing promoter to make it stronger (Zhou et al., 2014). mRNA translation can be blocked by using small RNAs (sRNA) and riboswitches system which express *cis* or *trans* encoded RNAs that hybridise with their target (Hu & Wang, 2018; Ueno et al., 2017).

Conventional methodology for genome engineering in cyanobacteria are largely dependent on the double-crossover or the single-crossover homologous recombination, using linear DNA or plasmid, through conjugation or transformation in naturally competent cyanobacterial strain (Vioque, 2007). The majority of cyanobacterial strains have multiple copies of their genome, therefore employing conventional strategies can be time-consuming and laborious to generate a homozygous strain in which all copies of a gene of interest present in the genome need to be mutated (Griese et al., 2011). The type-II CRISPR/Cas9 system from *Streptococcus pyogenes* has been found to be efficient for markerless and accurate genome editing of the cyanobacterial genome (Hsu et al., 2014). It induces a double-strand break in chromosomes which exerts selective pressure and thus increases the chance of integration of the gene of interest into all genome copies. This methodology increases the transformation efficiency in *S. elongatus* PCC7942 by approximately 23% compared to the conventional method (Li et al., 2016). The major obstruction of using Cas9 in cyanobacteria is its toxicity to the cells (Wendt et al., 2016), leading to the usage of CRISPR/Cpf1 or CRISPR/Cas12a which is a type V-A nuclease of the class II family of the CRISPR system (Ungerer & Pakrasi, 2016). It is first characterised in *Francisella novicida* and was found less toxic compared to Cas9 (Niu et al., 2019). The reports are available for the successful application of CRISPR/Cpf1 in the engineering of markerless, knock-ins, knock-outs, and point mutation in three model cyanobacterial species (Ungerer & Pakrasi, 2016).

For targeting essential genes or pathways, the CRISPR/Cas system has been modified further with deactivated nuclease domains. The dCas9 or dCas12a enzyme lacks the cleavage activity but maintain the ability to bind the target DNA along with sgRNA. The modified version of the CRISPR/Cas system is known as CRISPR-interference (CRISPRi). This system was first reported in *Synechocystis* sp. PCC6803 in which aldehyde reductase and dehydrogenase were knocked down (Yao et al., 2016). The modified dCas9 system achieved around 30% knock-down of the target gene (Gordon et al., 2016), while the dCas12a effectively repressed the target gene by 53–94% (Choi & Woo, 2020). Thus, the CRISPR system offers a promising tool for efficient and reliable genetic engineering in cyanobacteria.

## Challenges and Future Directions

While several advantages of using cyanobacteria for alkane production have been listed, quite a few challenges still need to be addressed to utilise its full potential. One of the major hurdles while manipulating the genome of cyanobacteria are the presence of multiple genome copies per cell (Watanabe, 2020), making it challenging to achieve genomic homogeneity, and requires multiple segregation steps to form a stable transformant line (Vavitsas et al., 2017). Nevertheless, the homologous recombination strategy offers a high probability of transformation by using an overlap of around 100 bp (Vogel et al., 2017). However, this needs a well-annotated genome with precise location and copy number of a gene of interest. Even with more efficient methods available, such as CRISPR which can generate markerless mutants, toxicity and off-target effects can limit the use and decrease the editing efficiency (Wendt & Pakrasi, 2019). Further, a problem arises while performing metabolic engineering to direct the flow of flux for improving the yield of required metabolite. Therefore, more insight into understanding the fundamentals of metabolic pathways and flux will be required to accelerate the desired pathway for enhanced yield.

One of the strategies to enhance alkane production is to engineer the AAR enzyme to increase its activity as it acts as a rate-limiting enzyme. Our group has recently shown the propensity of AAR to aggregate when overexpressed in *E. coli* (Sharma et al., 2021), suggesting the need for an improved AAR enzyme. In comparison with other model organisms such as *E. coli* and *S. cerevisiae*, limited genetic tools are accessible for manipulating the cyanobacterial genome. This leads to a slowdown of the basic research and development for using it as a chassis for useful biomolecules. However, efforts have been made to improve and simplify genetic manipulation for various purposes (Santos-Merino et al., 2019).

With the urgent need to improve our biofuel production capacity for significantly reducing the dependence on non-renewable sources of energy, there is a need for more investigation and attention to be given in the case of cyanobacteria. Through this review, we aimed to shed light on the basic machinery involved in alkane production in cyanobacteria and discuss progress that have been made to improve the alkane titre via homologous or heterologous pathway engineering. We also attempted to provide an insight into the development and application of the genetic tools in cyanobacteria. We hope that with the increase in further understanding of genetic information of cyanobacteria, it won't be too far when a commercially viable production of hydrocarbon will be made via microbial route.

## Data Availability

All relevant data are provided in the manuscript. Authors agree to provide any other data if requested.
